# A randomised controlled trial testing the efficacy of Fit after COVID, a cognitive behavioural therapy targeting severe post-infectious fatigue following COVID-19 (ReCOVer): study protocol

**DOI:** 10.1186/s13063-021-05569-y

**Published:** 2021-12-02

**Authors:** T. A. Kuut, F. Müller, A. Aldenkamp, E. Assmann-Schuilwerve, A. Braamse, S. E. Geerlings, K. B. Gibney, R. A. A. Kanaan, P. Nieuwkerk, T. C. Olde Hartman, D. Pauëlsen, M. Prins, K. Slieker, M. Van Vugt, C. P. Bleeker-Rovers, S. P. Keijmel, H. Knoop

**Affiliations:** 1 Department of Medical Psychology, Amsterdam University Medical Centers, University of Amsterdam, Amsterdam Public Health Research Institute, Amsterdam, The Netherlands; 2 Amsterdam University Medical Centers, Expert Center for Chronic Fatigue, Department of Medical Psychology, Vrije Universiteit Amsterdam, Amsterdam, The Netherlands; 3 Department of Lung Medicine, Catharina Hospital, Eindhoven, The Netherlands; 4 Department of Medical Psychology, Jeroen Bosch Hospital, Den Bosch, The Netherlands; 5 Department of Internal Medicine, Division Infectious Diseases, Amsterdam University Medical Centers, University of Amsterdam, Amsterdam Public Health Research Institute, Amsterdam, The Netherlands; 6 Victorian Infectious Diseases Service, Royal Melbourne Hospital, and Department of Infectious Diseases, University of Melbourne, at the Peter Doherty Institute for Infection and Immunity, Melbourne, Victoria Australia; 7 Department of Psychiatry, University of Melbourne, Austin Health, Heidelberg, Australia; 8 Department of Primary and Community Care, Radboud Institute for Health Sciences, Radboud University Medical Center, Nijmegen, The Netherlands; 9 Department of Infectious Diseases, Public Health Service of Amsterdam, Amsterdam, The Netherlands; 10 Department of Internal Medicine, Bernhoven, Uden, The Netherlands; 11 Division of Internal Medicine, Department of Infectious Diseases, Amsterdam University Medical Centers, University of Amsterdam, Amsterdam Institute for Infection and Immunity, Amsterdam Public Health Research Institute, Amsterdam, The Netherlands; 12 Department of Internal Medicine, Radboud university medical center, Nijmegen, The Netherlands

**Keywords:** Cognitive behavioural therapy, COVID-19, Post-Acute Sequelae of SARS-CoV-2 Infection (PASC), Post-COVID-19 syndrome, Long COVID, Long-Haul COVID-19 Fatigue, Internet therapy, Study protocol, Randomised controlled trial

## Abstract

**Background:**

Coronavirus disease 2019 (COVID-19) results in debilitating long-term symptoms, often referred to as Post-Acute Sequelae of SARS-CoV-2 Infection (PASC), in a substantial subgroup of patients. One of the most prevalent symptoms following COVID-19 is severe fatigue. Prompt delivery of cognitive behavioural therapy (CBT), an evidence-based treatment that has shown benefit in reducing severe fatigue in other conditions, may reduce post-COVID-19 fatigue. Based on an existing CBT protocol, a blended intervention of 17 weeks, *Fit after COVID*, was developed to treat severe fatigue after the acute phase of infection with SARS-CoV-2.

**Method:**

The ReCOVer study is a multicentre 2-arm randomised controlled trial (RCT) to test the efficacy of *Fit after COVID* on severe post-infectious fatigue. Participants are eligible if they report severe fatigue 3 up to and including 12 months following COVID-19. One hundred and fourteen participants will be randomised to either *Fit after COVID* or care as usual (ratio 1:1). The primary outcome, the fatigue severity subscale of the Checklist Individual Strength (CIS-fatigue), is assessed in both groups before randomisation (T0), directly post CBT or following care as usual (T1), and at follow-up 6 months after the second assessment (T2). In addition, a long-term follow-up (T3), 12 months after the second assessment, is performed in the CBT group only. The primary objective is to investigate whether CBT will lead to a significantly lower mean fatigue severity score measured with the CIS-fatigue across the first two follow-up assessments (T1 and T2) as compared to care as usual. Secondary objectives are to determine the proportion of participants no longer being severely fatigued (operationalised in different ways) at T1 and T2 and to investigate changes in physical and social functioning, in the number and severity of somatic symptoms and in problems concentrating across T1 and T2.

**Discussion:**

This is the first trial testing a cognitive behavioural intervention targeting severe fatigue after COVID-19. If *Fit after COVID* is effective in reducing fatigue severity following COVID-19, this intervention could contribute to alleviating the long-term health consequences of COVID-19 by relieving one of its most prevalent and distressing long-term symptoms.

**Trial registration:**

Netherlands Trial Register NL8947. Registered on 14 October 2020.

**Supplementary Information:**

The online version contains supplementary material available at 10.1186/s13063-021-05569-y.

## Background

The coronavirus disease 2019 (COVID-19) pandemic is a serious health crisis resulting in multiple symptoms in a substantial subgroup of patients. During acute COVID-19, up to 4 weeks after the onset of the infection [1], severe fatigue is one of the most prevalent symptoms [2–5]. When symptoms of COVID-19 continue for more than 12 weeks and are not explained by an alternative diagnosis, these symptoms are referred to as Post-Acute Sequelae of SARS-CoV-2 Infection (PASC), post-COVID-19 syndrome, long COVID or Long-Haul COVID [1, 6]. Concerns soon arose that severe fatigue might be one of the highly prevalent long-term consequences of the pandemic [7–9]. Indeed, in patients who required hospitalisation during acute COVID-19 [10, 11] and in non-hospitalised patients after mild COVID-19 [12, 13], 17–63% of the patients report fatigue 6-12 months following COVID-19 [10–13]. Research on other coronavirus infections like Severe Acute Respiratory Syndrome (SARS), Middle East Respiratory Syndrome (MERS) and other infectious diseases such as Q-fever indicate that around 20% of patients suffer from persistent post-infectious severe fatigue [14–16]. With the current 188 million confirmed COVID-19 cases globally [17], severe fatigue following COVID-19 might affect millions worldwide leading to long-term health problems, disability, and high illness-related costs. This calls for research into interventions addressing post-COVID-19 fatigue.

Fatigue in the acute phase of an infectious disease is most likely an adaptive response during the course of an infection [18]. Once the infection subsides, the fatigue decreases and most patients fully recover. However, in a subset of patients the fatigue persists. Post-infectious chronic fatigue is defined as severe fatigue persisting for more than 6 months following an infection, with detrimental effects on patients’ functioning, quality of life and societal participation [19]. It is unclear what causes the persistence of fatigue but several mechanisms have been suggested, such as a dysregulation of cytokines or neuro-inflammation [20, 21]. According to the cognitive behavioural model of post-infectious fatigue, the infection triggers fatigue and cognitive behavioural factors contribute to its perpetuation [18].

Research on other medical conditions has indeed shown that cognitive behavioural variables, like a disrupted sleep-wake pattern, low or unevenly distributed level of activity, or dysfunctional fatigue-related beliefs, can explain the persistence of fatigue [18, 22]. These factors seem transdiagnostic, i.e. they have been found to perpetuate fatigue across different diseases and long-term medical conditions [23, 24]. We expect that the same factors also play a role in fatigue following COVID-19. These cognitive behavioural perpetuating factors can be addressed in cognitive behavioural therapy (CBT) [25]. Previous research into post-infectious fatigue following Q-fever [26] and chronic fatigue in other conditions [27–30] has shown that CBT can lead to a significant reduction in fatigue severity and disability. Effect sizes are moderate to large with a substantial proportion of patients showing clinically relevant improvement in fatigue directly following treatment [26, 27, 29, 31]. We assume that addressing the transdiagnostic cognitive behavioural perpetuating factors may also lead to a reduction of severe fatigue following COVID-19. Data on long-term outcomes after CBT are mixed with some studies showing maintenance of treatment effects [32], while others show partial maintenance of treatment effects with relapse in a supgroup of patients [33–35].

The efficacy of CBT for fatigue has mostly been tested in populations with a long duration of fatigue and impairments, often several years. There is evidence that a long symptom duration is associated with a less favourable outcome of CBT [36]. Thus early intervention may help to prevent severe fatigue becoming chronic. On the other hand, in glandular fever, considerable spontaneous recovery from acute fatigue in the first three months after an infection [37] is observed. Three months following the acute phase of COVID-19 may therefore be the optimum time point to start an intervention.

The primary objective of the ReCOVer study is to investigate whether CBT results in a significantly lower mean fatigue severity score measured by the subscale fatigue severity of the Checklist Individual Strength (CIS-fatigue) [38] post-treatment and at 6 months follow-up as compared to care as usual. Secondary objectives are to determine whether CBT as compared to care as usual will result in (1) a smaller proportion of patients having severe fatigue, (2) a larger proportion of patients no longer being severely fatigued and reporting a clinically significant improvement, (3) a smaller proportion of patients having severe fatigue with a symptom duration of 6 months or longer, (4) improved physical functioning, (5) improved social functioning, (6) fewer somatic symptoms and (7) fewer problems concentrating. In an additional analysis, the long-term outcome of CBT (1 year post-treatment) will be investigated.

## Method

This is a two-arm, multi-centre randomised controlled trial (RCT) designed to compare the efficacy of 17 weeks of CBT on fatigue severity to that of care as usual. All participants are assessed at baseline (T0), post-CBT or care as usual (T1) and at follow-up six months after T1 (T2). Participants randomised to CBT will also be assessed at an uncontrolled long-term follow-up (T3), 12 months after completion of treatment.

In reporting the ReCOVer study, the SPIRIT (Standard Protocol Items: Recommendations for Interventional Trials) reporting guideline is used [39]. See Additional file 1 for the trial registration data set and Additional file 2 for the date and version identifier of the protocol.

### Trial search

Several trial registers were searched on 8th and 9th of July 2021. While various trials testing the efficacy of exercise therapy and rehabilitation on fatigue (as primary or secondary outcome) were identified, no trial has been registered testing the effect of CBT or another psychological intervention on fatigue following COVID-19.

### Recruitment 

Patients are recruited by medical professionals of participating hospitals in the Netherlands: Amsterdam University Medical Centers (Amsterdam UMC), location Academic Medical Center (AMC) and VU medical center (VUmc) in Amsterdam, Radboud university medical center (Radboudumc) in Nijmegen, Jeroen Bosch Hospital (JBZ) in Den Bosch, Catharina hospital in Eindhoven and Bernhoven in Uden. In addition, general practitioners (GP’s), public health services, patient organisations and the general public are informed about the study by flyers, leading to referrals via GP’s and self-referrals. For recruitment via self-referral, the study is advertised via social media, i.e. LinkedIn and Facebook, and adverts in local newspapers. Additionally, a study-website (www.moenacovid.nl) has been developed, where interested individuals can find information about the study, fill out a brief test to check whether they fulfil inclusion criteria, and find information on how to contact the research assistant.

### Participants

After (self)referral, interested patients are contacted by telephone by the research assistant to inform them about the study. All patients are screened for eligibility. The inclusion and exclusion criteria are shown in Table 1. For patients referred via a physician, the medical inclusion criteria (a and b) and exclusion criterion a (a somatic condiotion that can explain the fatigue) are checked by a physician or research nurse to ensure the patient has been diagnosed with COVID-19 and has no other psychiatric or somatic condition that can explain the fatigue. For self-referrals, these eligibility criteria are checked by contacting the patient’s GP with the permission of the patient. Self-referrers are also asked to send a copy or screenshot of their positive SARS-CoV-2 test result. In case the information in the medical record of the GP is insufficient to determine eligibility, self-referrals are sent to the outpatient clinic of the Amsterdam UMC (location AMC) or Radboudumc for screening. After that, they follow the procedure for inclusion of patients directly referred by a physician.

**Table 1:** Inclusion and exclusion criteria

Inclusion criteria	Exclusion criteria
a) Diagnosed with symptomatic COVID-19, confirmed by a positive PCR for SARS-CoV-2 *or* another positive NAAT test (RT-PCR, LAMP, TMA or mPOCT) *or* positive SARS-CoV-2 serology *or* a positive Antigen test *or* CORADS 4 or 5 on CT-scan. b) Three up to including 12 months after being diagnosed with COVID-19 or after hospital discharge in case the patient was admitted. c) Severe fatigue, operationalised as a score of ≥ 35 on the subscale fatigue of the Checklist Individual Strength (CIS) [38]. Fatigue started with or increased substantially directly after the onset of symptoms of COVID-19, as reported by the patient and confirmed by their GP or treating physician. d) Limitations in physical functioning operationalised as a score of ≤ 65 on the Short Form Health Survey (SF-36) [40] or social disability operationalised as a score of ≥ 10 on the Work and Social Adjustment Scale (WSAS) [41]. e) Age of 18 years or older. f) Sufficient command of the Dutch language.	a) Known psychiatric or somatic condition that can explain the fatigue. Screening for somatic condition is done by the referring physician or the patient’s GP in case of self-referral. Participants are screened for the presence of post-traumatic stress disorder (PTSD) with the PTSD Checklist for DSM-5 (PCL-5) [42] and for the presence of depressive disorder with the Beck Depression Inventory for Primary Care (BDI-PC) [43, 44]. When the score on the BDI-PC is ≥ 4 or the score on the PCL-5 is ≥ 33, the Mini-International Neuropsychiatric Interview (M.I.N.I.) [45] is conducted to determine if patients meet the criteria of PTSD or a depressive disorder. b) Current participation in a multi-disciplinary rehabilitation programme aimed to ameliorate the consequences of COVID-19. c) Objective hypoxaemia in rest for which oxygen therapy at home is indicated.

After obtaining informed consent, online screening questionnaires are administered by the researcher to verify the remaining eligibility criteria (see Table 1). The subscale fatigue severity of the CIS-questionnaire [38] is used for inclusion criterion c, to screen for the presence of severe fatigue. For inclusion criterion d, the presence of limitations in physical or social functioning, the subscale physical functioning of the Short Form Health Survey (SF-36) [40] and the Work and Social Adjustment Scale (WSAS) [41] are used. Participants are screened for the presence of post-traumatic stress disorder (PTSD) with the PTSD Checklist for DSM-5 (PCL-5) [42], and for the presence of a depressive disorder with the Beck Depression Inventory for Primary Care (BDI-PC) [43, 44] (exclusion criterion a, i.e. the presence of a psychiatric condition that can explain the fatigue). If the score on the BDI-PC is ≥ 4 or the score on the PCL-5 is ≥ 33, the Mini-International Neuropsychiatric Interview (M.I.N.I.) [45] is conducted by a trained research assistant via phone to determine if patients meet the criteria of a depressive disorder or PTSD. In that case, patients will not be included. Instead, the GP is contacted for referral to appropriate treatment. If two persons from the same household are screened and found to be eligible, only one of them can participate (based on their choice). This is to prevent contamination. The other person is allocated to the same condition outside the study context. If a patient has been infected twice, the first diagnosis with COVID-19 or hospital discharge must be from 3 months up to and including 12 months ago (inclusion criterion a). Only in those cases where fatigue commenced or increased substantially directly after the second infection, the second infection is used as the reference point to determine eligibility.

### Changes in trial design following approval

At the beginning of the study, the upper limit for including patients was extended from initially 6 months to 12 months after diagnosis or hospital discharge (inclusion criterion b). This enhanced the feasibility of the project as patients from the first COVID-19 wave in the Netherlands were then eligible when initiating recruitment in October 2020.

Inclusion criterion a, symptomatic and confirmed COVID-19, was adapted following the introduction of new SARS-CoV-2 tests. Part of original criterion a, typical symptoms and being part of a household in which another person tested positive for SARS-CoV-2 by PCR 2 weeks before or after the first day of illness, was dropped because of increased test capacity. No patients were included based on this criterion.

### Procedure 

**Fig. 1:**
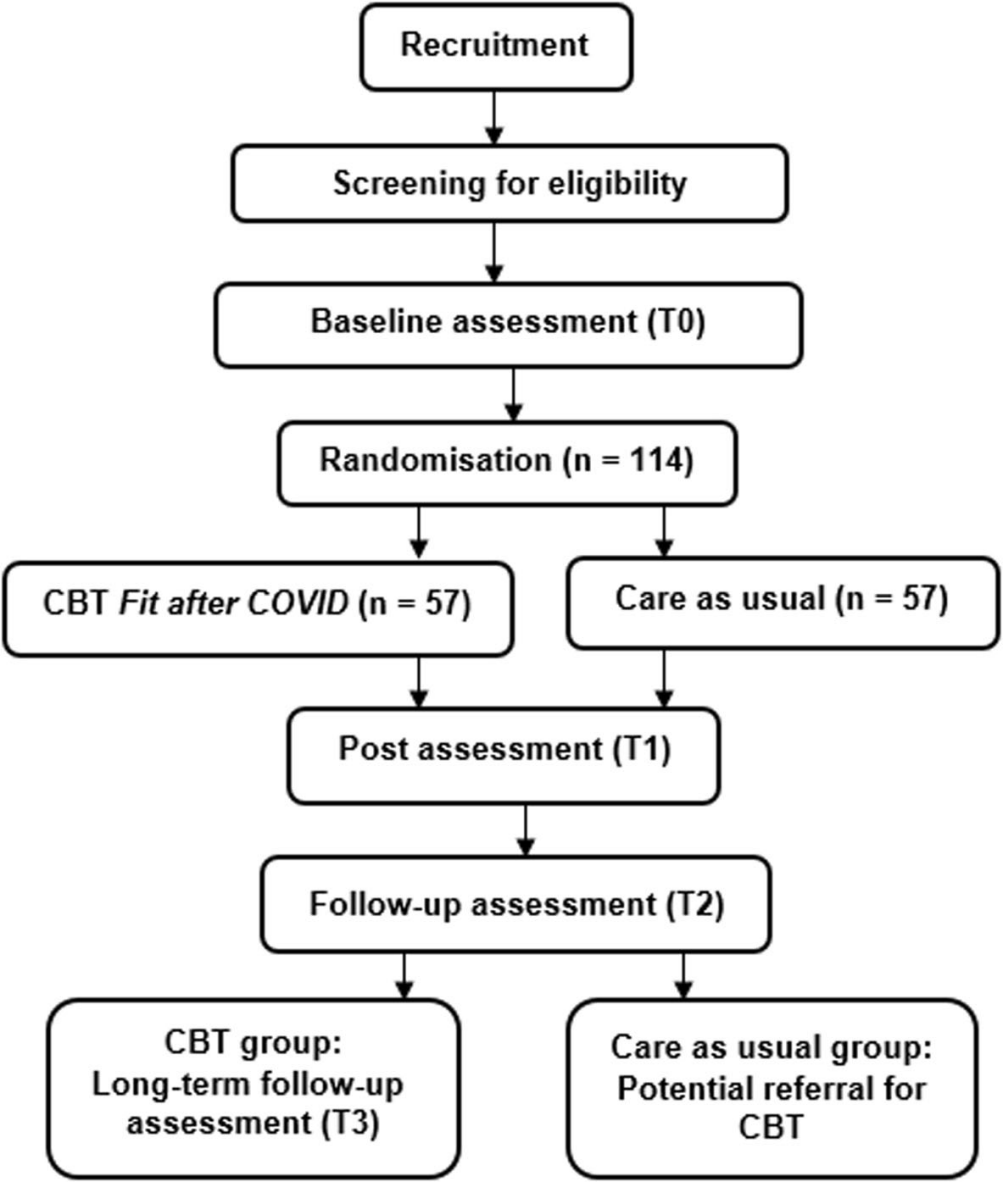
ReCOVer flow-chart

Eligible and consenting participants start with the baseline assessment (T0). Following T0, participants are randomised to the CBT group or care as usual. In case of randomisation to CBT, the first treatment session is planned within 2 weeks after randomisation. Nineteen weeks after randomisation all participants are assessed again (T1). For participants assigned to CBT this is the post-intervention assessment. At follow-up (T2), 6 months after T1, all participants are assessed again. Participants randomised to the CBT group are also assessed at long-term follow-up (T3), 12 months after treatment. Due to ethical considerations, participants from the care as usual arm are offered referral for existing CBT for chronic fatigue after the follow-up assessment (T2) and hence, cannot serve as a control for the long-term follow-up assessment. A flow-chart of the study design is shown in Fig. 1.

### Intervention: Fit after COVID

CBT for severe fatigue has been found to be effective in several RCT’s [27, 29, 30]. The efficacy of the internet version of this treatment protocol is also confirmed [29]. The adapted version targeting fatigue after COVID-19 is named *Fit after COVID *(in Dutch*: Fit na COVID).* The main part of the intervention is based on an existing CBT manual for Chronic Fatigue Syndrome (CFS) by our research group [46] and was adapted by experienced cognitive behavioural therapists (HK, TK). As the original CBT manual for CFS, *Fit after COVID* is based on a cognitive behavioural model of fatigue [26]. According to this model, a disease or stressor (here: COVID-19) initially triggers fatigue while cognitive behavioural variables perpetuate fatigue. The seven perpetuating factors addressed in *Fit after COVID* are (1) disrupted sleep-wake pattern, (2) dysfunctional beliefs about fatigue, (3) low or unevenly distributed level of activity, (4) perceived low social support, (5) problems with processing the acute phase of COVID-19, (6) fears and worries regarding COVID-19, and (7) poor coping with pain.

In the adapted version, the psychoeducation and the explanation about perpetuating factors now consistently refer to COVID-19 as the trigger of the fatigue. The modules on processing the acute phase of COVID-19 and fears and worries regarding COVID-19 were adapted from a treatment manual for fatigue in cancer survivors (i.e. coping with cancer and cancer treatment, and fear of cancer recurrence) [47].

Three patients suffering from the long-term effects of COVID-19, recruited by the patient organisation *Lung Foundation Netherlands*, read the text of the treatment protocol and tested the online modules. They evaluated the content, text and usability of the internet platform very positively.

*Fit after COVID* is available as a blended internet intervention, but can also be provided in face-to-face format. There is no difference in content between the two formats. The treatment is delivered by cognitive behavioural therapists and starts with an intake session, if possible face-to-face, with the assigned therapist. If the participant cannot or does not want to travel to the hospital, the intake can take place via a secure video connection. The remaining treatment can be followed online on a secure platform where participants have access to their modules. They are supported by their therapist who provides feedback via e-mail or during video calls or face-to-face on the progress they made. The planned duration of the CBT is 17 weeks.

*Fit after COVID* consists of up to nine modules: an introductory module, an evaluation module and seven modules which address fatigue-perpetuating factors. All participants start with setting treatment goals in module 1. Then, all participants follow the three core modules on the perpetuating factors of disrupted sleep-wake pattern, low or unevenly distributed level of activity and dysfunctional beliefs about fatigue. The four optional modules are targeting perceived low social support, problems with processing the acute phase of COVID-19, fears and worries regarding COVID-19 and poor coping with pain. All participants complete the treatment by working on their treatment goals in module 9.

The intervention *Fit after COVID* is personalised in two ways. First, the content of the core modules is adapted to the participant based on the baseline assessment. The module on disrupted sleep-wake pattern is adapted based on the sleep diary and the Insomnia Severity Index (ISI) [48]. The module on dysfunctional beliefs about fatigue is adapted based on questionnaires assessing specific dysfunctional beliefs, i.e. catastrophizing assessed by the Jacobson-Fatigue Catastrophizing Scale (J-FCS) [49], focussing on fatigue assessed by the Illness Management Questionnaire (IMQ) [50] and low self-efficacy assessed by the Self-Efficacy Scale (SES) [51, 52]. The module on graded activity contains a version for relatively active participants and a version for low active participants, based on the activity pattern assessed with an actigraph [53]. Participants with low activity pattern immediately start with a gradual increase in their daily physical activity, while participants with a relative active activity pattern learn first to evenly distribute their activities during the day and then subsequently gradually increase their daily activity. As the second way to personalize the treatment, participants only follow the optional modules aimed at fatigue-perpetuating factors that apply to them. The modules are selected based on their scores on baseline questionnaires and on information collected by the therapist during the intake session. Questionnaires indicating whether optional modules are relevant are the Van Sonderen Social Support Inventory Discrepancy scale (SSL-D) and Interactions scale (SSL-I) [54, 55] for the module on perceived low social support, the Impact of Event Scale (IES) [56, 57] for the module on problems with processing the acute phase of COVID-19 and the subscale pain of the SF-36 [40] for the module on poor coping with pain. For the selection of the module on fears and worries regarding COVID-19, the Cancer Worry Scale (CWS) [58] was adapted to the COVID-19 Worry Scale (COWS).

See Table 2 for an overview and description of each module, the assessment tools and accompanying cut-off points.

**Table 2:** Overview of *Fit after COVID* modules

Module name	Instrument and cut-off score	Brief description of module content
1. Goal setting		- Psychoeducation regarding the cognitive behavioural model of post-infectious fatigue following COVID-19. - Patients set treatment goals in concrete activities that will be performed when the patient’s impairments and fatigue are alleviated.
2. Sleep-wake pattern	Sleep diary ISI ≥ 10	- Targets a disrupted sleep-wake pattern. - Patients establish a regular sleep-wake pattern and follow sleep-hygiene practices. - Patients are encouraged to stop sleeping or lying down at daytime.
3. Helpful thinking	J-FCS ≥ 16 IMQ ≥ 30 SES ≤ 19	- Targets dysfunctional cognitions regarding fatigue. - Patients learn to identify unhelpful thoughts and replace them with helpful thoughts, gain more self-efficacy and learn to focus less on their fatigue. - Patients learn to redirect their attention away from bodily symptoms.
4. Social support ^a^	SSL-D ≥ 14 SSL-I ≥ 50	- Targets low perceived social support and negative interactions. - Patients learn how to communicate with significant others about their fatigue, be assertive and adapt expectations about their environment.
5. Graded activity	Activity pattern (actigraph), relatively active vs. low active	- Targets a low or fluctuating physical activity pattern. - Patients with low activity pattern start with gradual increase in their daily physical activity. - Patients with a relative active activity pattern learn first to evenly distribute their activities during the day and then subsequently gradually increase their daily activity.
6. Processing the acute phase of COVID-19 ^a^	IES, subscales ≥ 10	- Targets emotional problems of patients who did not process the acute phase of COVID-19. - Patients are helped to process negative experiences from the acute phase of their illness.
7. Fear and worries regarding COVID-19 ^a^	COWS ≥ 10	- Targets excessive fears and worries regarding COVID-19. - Patients record what the content of their fear and worries is regarding COVID-19. - Patients learn to formulate helpful thoughts and to distance themselves from their anxious thoughts.
8. Coping with pain ^a^	Subscale pain of the SF-36 ≤ 40	- Targets dysfunctional cognitions with respect to pain. - Patients are helped to deal with pain in such a way that it does not limit them during the gradual increase of activities.
9. Realising goals		- Patients make an action plan to work on their formulated treatment goals, like increasing social and mental activities. - Patients learn about the difference between severe fatigue and normal fatigue. - Patients learn to let go of the regular sleep-wake pattern and even distribution of activities. - Patients evaluate their progress.

^a^Optional module

*ISI* Insomnia Severity Index [48], *J-FCS* Fatigue Catastrophizing Scale [49] *IMQ* Illness Management Questionnaire [50] *SES* Self-Efficacy Scale [51, 52], *SSL-D* Van Sonderen Social Support Inventory, subscale discrepancy, *SSL-I* Van Sonderen Social Support Inventory, subscale interactions [54, 55], *IES* Impact of Event Scale [56, 57], *COWS* COVID-19 Worry Scale (adapted from Cancer Worry Scale (CWS)) [58], *SF-36* Short Form Health Survey [40]

### Therapist training

Six therapists working at the participating hospitals completed a 4-day training programme provided by a senior clinical psychologist (HK). The training consists of discussing each treatment module and practising the interventions in role-playing with simulation patients, being professional actors. An introduction to the online platform, exercises in writing e-mails and giving online feedback are part of the training. Five therapists working at the Expert Centre for Chronic Fatigue (ECCF) of the Amsterdam UMC were already trained in the protocol for CBT for CFS, but followed an additional 2-hour training programme on COVID-19-specific issues.

### Adherence and treatment integrity

All therapists are supervised bi-weekly by an experienced clinical psychologist (HK, TK) to ensure treatment integrity. In this supervision, all cases are discussed. Therapists register each face-to-face, video, phone and e-mail contact with the participant. The duration of the contact and treatment modules delivered are registered. All activities of the participant and therapist on the platform are registered via log data and are available for analysis.

### Care as usual

After randomisation, participants in the care as usual condition have no access to *Fit after COVID* during the study, but are not restrained from using any form of care for fatigue or other COVID-19 related symptoms. Care as usual for post-COVID-19 patients can entail follow-up contacts with their treating physician or GP, physical training, occupational therapy or rehabilitation. At T1, all participants are questioned about the care they received since baseline assessment. Participants who are still severely fatigued, i.e. a score of ≥ 35 on the CIS-fatigue at T2, and voice a care need can be referred for CBT for severe fatigue; this will be provided by the ECCF of the Amsterdam UMC.

### Randomisation

Allocation of participants to either CBT or care as usual is stratified based on (1) illness severity during the acute stage of COVID-19 (no admission to hospital; admitted to hospital, not on the intensive care unit (ICU); ICU during hospitalisation) and (2) dyspnoea based on the Medical Research Council (MRC) [59] score (< 3 vs. ≥ 3). This is because dyspnoea might influence the response to CBT by interfering with the graded activity module. A web-based randomisation programme in Castor EDC [60] is used to generate the randomisation sequence. The allocation ratio is 1:1. Block-randomisation with randomly selected block sizes (i.e. 2, 4 or 6) is used. The randomisation is performed by a research assistant in the presence of the participant who is on the phone. The research assistant, the researcher and the participants are blinded to the allocation sequence. Due to the nature of the intervention, blinding to allocation is not feasible. Data will be analysed by an independent statistician using a data file which is blinded for treatment allocation.

### Outcome measures

Outcome measures consist of self-reported questionnaires, which are assessed at T0, T1, T2 and T3. See Table 3 for the measurements at all time points. The questionnaires are administered online using Castor EDC [60]. Participants receive an e-mail with a personal link to fill out the questionnaires. For optimal retention, participants are contacted by the research assistant if they have not completed the questionnaires within one week. When participants are not willing to complete all measurements at T1, T2 and/or T3, they are asked to at least fill out the CIS-fatigue [38], which is the primary outcome measure. At T0 and T1, data on physical activity level and sleep are also gathered by using an actigraph and completing a sleep diary.

**Table 3:**
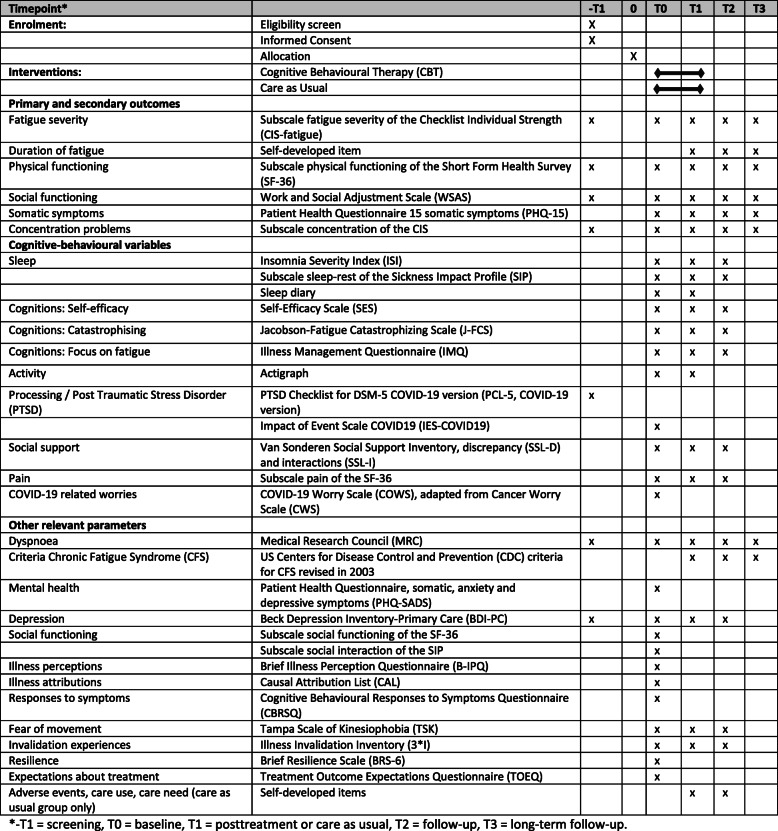
Measurements and time points

### Primary outcome

*Fatigue severity* is assessed by the subscale fatigue severity of the CIS [38]. The CIS consists of 20 items scored on a 7-point Likert scale and measures four dimensions of fatigue, i.e. fatigue severity, concentration problems, motivation and activity. The CIS-fatigue, the primary outcome, consists of eight items, each item is scored on a 7-point Likert scale. The range of scores is 8 to 56, with a higher score indicating more severe fatigue. The cut-off score for severe fatigue is ≥ 35 [38] on the CIS-fatigue. Previous research supports the reliability and validity of the CIS [38, 61, 62].

### Secondary outcomes

*Limitations in physical functioning* are assessed by the subscale physical functioning of the SF-36 [40] which consists of ten items scored on a 3-point scale. The weighted subscale score ranges from 0 to 100, with a higher score indicating less limitations. A score of ≤ 65 is indicative of substantial limitations in physical functioning [63]. The SF-36 is a reliable and valid instrument [64].

*Social disability* is assessed by the WSAS [41, 65] which consists of five items assessing different domains of functioning, e.g. work and social leisure activities, scored on an 8-point Likert scale. A score of ≥ 10 is indicative of significant functional impairment [41, 65]. The WSAS is a reliable and valid instrument [41, 65.

*Somatic symptom severity* is assessed by the somatic symptoms scale of the Patient Health Questionnaire (PHQ) [66]. The PHQ-15 consists of 15 items referring to somatic symptoms. Each symptom is scored on a 3-point scale with scores of 5, 10 and 15 indicate low, medium, and high symptom severity. Research supports the reliability and validity of the PHQ-15 [66].

*Problems concentrating* are assessed by the subscale concentration of the CIS which consists of five items scored on a 7-point Likert scale. The scores range from 5 to 35, with a higher score indicating more difficulties in concentrating.

### Demographics and patient characteristics

The demographic characteristics include age, sex, relationship/cohabitation status, level of education and employment status. Patient characteristics include lenght, weight, morbidities, hospital/ ICU admission, start date of COVID-19 symptoms, date of COVID-19 test, type of COVID-19 test, physician visit for COVID-19 and vaccination status. All of the characteristics are assessed by self-report during screening. Vaccination status is also assessed at T1.

### Other study outcomes

*Activity:* An actigraph is used to assess the participant’s level of physical activity. The actigraph is worn around the wrist for 14 consecutive days and nights for a reliable estimate of daily activity for 12 full days. The actigraph has been shown to be a reliable and valid instrument for the assessment of physical activity [53, 67].

*Sleep parameters:* During the 14 days participants wear the actigraph, a sleep diary [68] is also completed daily, if possible within one hour of getting out of bed in the morning. Participants have to fill in (1) the time they go to bed (in hours and minutes), (2) the time they try to fall asleep, (3) the sleep onset latency, (4) time awake at night, (5) time out of bed, (6) time of waking-up, (7) time of getting out of bed and (8) a rating of the sleep quality (scale 0–10) [69]. Sleep problems are also assessed by the ISI [48] and the subscale sleep-rest of the Sickness Impact Profile (SIP) [70, 71].

*Fatigue-related behaviours, cognitions and emotions*: Based on the cognitive behavioural model for fatigue in CFS and fatigue in other long-term conditions, several fatigue-related behaviours, cognitions and emotions are assessed. These are dysfunctional beliefs assessed by the J-FCS [49], IMQ [50] and the SES [51, 52, social support assessed by the SSL-D and SSL-I [54, 55], problems with processing assessed by the IES [56, 57], and pain assessed by the subscale pain of the SF-36 [40]. To assess COVID-19 related worries, we adapted the Cancer Worry Scale [58]. The resulting COVID-19 Worry Scale (COWS) consistently refers to COVID-19 instead of cancer. For participants randomised to CBT, the T0 actigraph data, sleep data and questionnaires about fatigue-related behaviours, cognitions and emotions are used to tailor *Fit after COVID* to the individual (see Intervention paragraph).

*Other relevant parameters:* The criteria for CFS are assessed by the US Centers for Disease Control and Prevention (CDC) criteria revised in 2003 [72]. Mental health is assessed by the patient health questionnaire, somatic, anxiety and depressive symptoms (PHQ-SADS) [73]. Social functioning is assessed by the subscale social functioning of the SF-36 [40] and the SIP [71]. Illness perceptions are assessed by the Brief Illness Perception Questionnaire (B-IPQ) [74, 75]. Illness attributions are assessed by the Causal Attribution List (CAL) [76]. Responses to symptoms are assessed by the Cognitive Behavioural Responses to Symptoms Questionnaire (CBRSQ) [77, 78]. Fear of movement is assessed by the Tampa Scale of kinesiophobia (TSK) [79]. Invalidation experiences are assessed by the Illness Invalidation Inventory (3*I) [80]. Resilience is assessed by the Brief Resilience Scale (BRS-6) [81, 82]. Expectations about treatment are assessed by the Treatment Outcome Expectations Questionnaire (TOEQ) [83]. All study parameters are assessed as shown in Table 3.

### Primary and secondary objectives

The primary objective is to investigate whether CBT will lead to a significantly lower mean fatigue severity score measured with the CIS-fatigue across follow-up visits T1 and T2 as compared to care as usual.

Secondary objectives are to investigate whether CBT as compared to care as usual will result in:

- A smaller proportion of patients meeting the cut-off score for severe fatigue (i.e. caseness as defined by a CIS-fatigue score of ≥ 35) at follow-up assessments T1 and T2 separately;
- A higher proportion of patients no longer meeting the cut-off score (as defined in a) and additionally, reporting a clinical significant change (i.e. Reliable Change Index (RCI)) [84] in fatigue severity at follow-up assessments T1 and T2 separately;
- A smaller proportion of patients with chronic fatigue (i.e. caseness as defined in a) and self-reported duration of fatigue of 6 months or longer at T1 and T2 separately;
- Significantly higher mean physical functioning as assessed with the SF-36 across follow-up assessments (T1 and T2);
- Significantly lower mean level on the WSAS across follow-up assessments (T1 and T2);
- Significantly lower mean level of somatic symptoms as assessed with the PHQ-15 across follow-up assessments (T1 and T2); and
- Significantly lower mean level of cognitive symptoms as assessed with the subscale concentration problems of the CIS across follow-up assessments (T1 and T2).

### Adverse events (AE’s)/serious adverse events (SAE’s)

At T1, all participants are asked to report if they experienced new symptoms or an increase in existing symptoms during CBT or care as usual. All AE’s reported by the participant or observed by the therapists or research staff are recorded and reported.

### Handling and storage of data and documents

Personal data is handled confidentially and is coded in compliance with the EU General Data Protection Regulation Act (in Dutch: *Uitvoeringswet* AVG, UAVG). Identifying patient information is coded for all study procedures. Only the PI (HK) and one researcher (TK) are allowed to access the keyfile with both participant codes and identifying participant data. The data collected with the actigraph is stored and processed locally on the secure servers of the Amsterdam UMC. The sleep diary is completed by the participants on a paper-and-pencil sheet. This sheet does not include personal information but only the patient’s identification code. The data cannot be traced back to specific participants in reports and publications on the study. Data will be stored at the Department of Medical Psychology of the Amsterdam UMC, location AMC, for 15 years following completion of the project. Data of *Fit after COVID* on the online platform will also be stored for 15 years on a secure server.

### Monitoring and auditing

This study is subject to on-site monitoring in accordance with the quality assurance advice of the Dutch Federation of University Medical Centres regarding research involving human subjects [85]. On-site monitoring is based on the risk classification negligible. The investigator will submit a summary of the progress of the trial to the ethics committee once a year. Information will be provided on the date of inclusion of the first subject, numbers of subjects included, numbers of subjects that have completed the trial, SAEs and amendments.

### Ethics approval and consent to participate

This study was reviewed and approved by the medical ethics committee of the Amsterdam UMC, location AMC (registration nr. 2020_182, NL74828.018.20) and by the local ethics committees of the participating hospitals. The study is registered in the Netherlands Trial Register (NTR) (NL8947). The NTR is updated in case of changes to the study protocol. Written informed consent will be obtained from all participants by the researcher (TK) and the research assistant (DP). All changes in the protocol are sent for approval to the medical ethics committee.

### Participant withdrawal

Participants can leave the study at any time for any reason without any consequences. The reasons are documented. The researcher can withdraw a participant from the study in case of incorrect enrolment. Participants are informed of their possibility to withdraw from the study during the intake and in the information letter. Despite leaving or being withdrawn from the study, treatment can be continued. The investigator, the treating physician, or the treating psychologist can decide to withdraw a participant from the study for urgent (medical) reasons. These reasons are documented. Randomised participants who drop out of the study are not replaced.

### Sample size calculation

The sample size calculation is based on testing the primary hypothesis that CBT leads to a significantly lower mean fatigue severity score (CIS-fatigue) across the first two follow-up assessments (T1 and T2) as compared to care as usual. A difference of 6 points on the CIS-fatigue is considered clinically relevant [38]. Based on previous research we assume a common standard deviation of 12 and correlation coefficients of 0.4 among CIS-fatigue scores assessed at T1 and T2 [27, 31].

With a sample size of 45 in group 1 (CBT) and 45 in group 2 (care as usual), a two-sided test for the time averaged difference between two means in a repeated measures design with a 0.05 significance level has 80% power to detect a difference in means of 6 in a design with 2 repeated measurements when the standard deviation is 12 and the between-level correlation is 0.4. Assuming a conservative dropout of 20%, we will randomise 114 participants, 57 to each condition.

### Statistical analyses

Descriptive statistics (i.e. participant characteristics) will be presented in tables, with separate columns for participants randomised to CBT vs. participants randomised to care as usual. Categorical variables (e.g. sex, relationship status) will be presented in participant numbers (*n*) and percentages (%). Continuous variables (e.g. age, CIS-fatigue baseline score) will be presented in means and standard deviations (SD) or, if appropriate due to skewness of data, as median with its interquartile range. Participant numbers, dropout and reasons for dropout will be presented in the study flow-chart. In all analyses, two-sided *p* values of < 0.05 are considered to indicate statistical significance. All analyses will be conducted in SPSS.

Change in fatigue scores between T0 and T1 for each individual participant will be shown in a figure using the Leeds RCI calculator [86]. This figure will show whether the change was clinically significant and reliable, or reliable only, and if there was no change or a deterioration in fatigue.

The primary study parameter will be analysed according to intention-to-treat, i.e. all randomised participants will be included in the analysis and they are analysed in the group to which they were randomly allocated. The primary hypothesis will be tested using a mixed linear model. The model will include T1 and T2 fatigue severity score as a dependent variable, condition (CBT vs. care as usual), time (T1, T2), condition by time interaction as fixed effects, a random intercept and the fatigue severity score at baseline as covariate. It will be examined if the main effect of condition from the mixed linear model, i.e. the mean difference in CIS-fatigue scores across T1 and T2 while controlling for baseline fatigue, is statistically significant. If the main effect of condition is found to be statistically significant, the statistical significance of the between group differences at the separate time points (T1 and T2) will be interpreted.

Cohen’s d effect sizes will be calculated by dividing the parameter estimate for the mean difference in CIS-fatigue scores between both conditions from the mixed linear model by the pooled standard deviation of both conditions combined. Effect size magnitudes will be interpreted as small (0.2 to 0.5), medium (0.5 to 0.8) and large (greater than 0.8) [87].

To explore the robustness of our findings from the primary analysis, four sensitivity analyses will be conducted. First, per protocol analysis will be conducted. Treatment completion will be operationalized in two ways, i.e. by including (a) participants who have filled out the treatment goals and opened the standard modules of the intervention and (b) participants who have completed the intervention according their therapist.

Second, as the primary outcome consists of the mean CIS-fatigue score across T1 and T2, the extent to which our result would change if based on a single time point will be explored. Therefore, the analysis with the CIS-fatigue score at T1 as a dependent variable and with the CIS-fatigue score at T2 as a dependent variable adjusted for CIS-fatigue score at baseline using analysis of covariance will be repeated. For each of the time points Cohen’s d effect sizes will be calculated.

Third, the extent to which dyspnoea at T0, disease severity, time since diagnosis of COVID-19, age and sex have an impact on the primary outcome will be explored, and whether this impact differs between CBT and care as usual. These analyses will be conducted by including these variables and their interaction-terms with condition as covariates in the mixed linear models.

Fourth, missing values will be replaced with multiple imputation using chained equations and the data will be reanalysed. The imputation model will include all baseline sociodemographic and clinical characteristics assessed at baseline and follow-up measurements. A total of five data sets will be imputed and pooled according to Rubin’s rule.

Secondary outcomes will also be analysed according to intention-to-treat. For T1 and T2, it will be determined if there is a clinical meaningful improvement in fatigue, defined as a RCI [84] of more than 1.96 and a decrease of the fatigue level to a normal range (i.e. a score of < 35 on the CIS-fatigue). For secondary outcomes that are dichotomous, separate logistic regression analysis will be done for T1 and T2. For secondary outcomes that are continuous (i.e. physical functioning, work and social adjustment, somatic symptoms, cognitive symptoms), differences between CBT and care as usual will be analysed using mixed linear models as described above.

For the long-term follow-up (T3) examining the course of fatigue over time within the CBT group, linear mixed model analyses will be applied, where the depended measure CIS-fatigue is assessed at T0, T1, T2 and T3.

## Discussion

The ReCOVer trial outlined in this article is the first RCT testing CBT targeting post-COVID-19 fatigue. The trial determines whether CBT, called *Fit after COVID*, is effective in reducing severe post-COVID-19 fatigue. Secondary outcomes investigated are the proportion of patients no longer being severely fatigued (operationalised in different ways), changes in physical and social functioning, and changes in the number and severity of somatic symptoms and problems concentrating.

If proven efficacious, CBT could contribute to alleviating the long-term health consequences of the pandemic by reducing one of its most prevalent and distressing symptoms affecting millions of people worldwide. Additionally, in the current pandemic where most people with symptoms are tested, COVID-19 is diagnosed early and the onset of the infection in known. This, together with the large number of patients, provides a unique opportunity to promptly intervene and prevent the development of chronic post-infectious fatigue. In chronic fatigue, a long symptom duration is associated with a less favourable outcome of CBT [33]. Therefore, timely intervention may not only prevent substantial health decline and societal costs but also help in the sustainment of gains made during treatment. In the ReCOVer study the upper limit for including patients was extended from initially 6 months to 12 months after COVID-19 diagnosis or hospital discharge. Despite this extension, we are still able to test whether we can prevent the development of chronic fatigue. Although a duration of 6 months is generally used to define chronic fatigue, this cut-off is somewhat arbitrary. Also in cases where fatigue is present since 12 months after COVID-19 it can be considered as a relatively short duration compared to previous intervention studies where patients have been included with an average fatigue duration up to 5 years [29, 63].

The online delivery of CBT has several advantages over face-to-face delivery: (1) it ensures therapy continuation despite preventive public health measures, (2) the intervention is easily accessible, (3) it reduces patient burden as it saves time and costs to travel to the treatment centre and (4) for therapists, less time investment is needed which increases treatment capacity [27, 29]. It has been shown that CBT interventions for severe fatigue can be successfully implemented in other countries and health care systems [88, 89]. These advantages would ensure that the intervention can be readily implemented, reaching a large group of patients.

A follow-up 12 months after completing CBT will be conducted to determine the long-term effect of CBT for fatigue after COVID-19. Data on long-term outcomes after CBT are mixed [32–35]. If the effect of *Fit after COVID* is found to abate over time, this might be a reason to expand the CBT with booster sessions. As described, a limitation of this design is that the third follow-up assessment is limited to participants in the CBT arm of the RCT and thus is not controlled. 

Additionaly, our intervention targets cognitive behavioural factors known to perpetuate severe fatigue. Other mechanisms such as a dysregulation of cytokines or neuro-inflammation [20, 21] or other physical consequences of COVID-19 like a compromised lung function are not addressed, but may also be (partly) responsible for the ongoing fatigue. More research may elucidate if certain subgroups of patients can or cannot benefit from CBT [90]. Relatedly, our search of trial registers showed that various trials testing the efficacy of exercise therapy and rehabilitation on post-COVID-19 fatigue are currently conducted. Based on evidence on the effect of exercise on severe fatigue in other conditions [91, 92], also this approach seems promising to relieve post-COVID-19 fatigue. Another limitation of our study is the exclusive focus on fatigue. While fatigue is the most reported symptom in patients with long COVID [93], most patients also report other symptoms.

As COVID-19 is a new disease and diagnostic procedures and treatment are evolving quickly, our inclusion criteria and measurements have been updated since the trial started, i.e. the upper limit of time between COVID-19 and enrolment has been extended and new SARS-CoV-2 tests have been added to the inclusion criteria.

In sum, this is the first trial testing the efficacy of CBT on severe post-COVID-19 fatigue. If found to be effective, it may help to relieve one of the most prevalent and distressing post-COVID-19 symptoms. Its online format fosters easy and widespread implementation which could help reach millions of people affected worldwide.

## Trial status

Patient recruitment started in November 2020 with version 3 of the protocol. At the time of submission, 89 participants have been randomly assigned. The trial is being conducted in accordance with the protocol version 4, dated 21 April 2021. Completion of inclusions is expected in October 2021.

## Supplementary Information

**Additional file 1: Table S1.** World Health Organization (WHO) Trial Registration Set.

**Additional file 2:** Date and version identifier of the protocol.

**Additional file 3:** Informed consent form (in Dutch).

## Data Availability

Any request to share the data of this RCT will be considered by the trial steering committee and will need to be approved by the ethics committee of the Amsterdam UMC, location AMC. An informed consent form in Dutch is available as a supplement.

## Abbreviations

AE: Adverse event
BDI-PC: Beck depression inventory for primary care
CBT: Cognitive behavioural therapy
CFS: Chronic fatigue syndrome
CIS: Checklist individual strength
CORADS: COVID-19 reporting and data system
COVID-19: Coronavirus disease 2019
CWS: Cancer worry scale
COWS: COVID-19 worry scale
ECCF: Expert centre for chronic fatigue
GP: General practitioner
ICU: Intensive care unit
IES: Impact of event scale
IMQ: Illness management questionnaire
ISI: Insomnia severity index
J-FCS: Jacobson-fatigue catastrophizing scale
LAMP: Loop mediated isothermal amplification
MERS: Middle East respiratory syndrome
M.I.N.I.: Mini-international neuropsychiatric interview
mPOCT: Moleculaire point of care test
NAAT: Nucleic acid amplification test
PASC: Post-acute sequelae of SARS-CoV-2 infection
PCL-5: PTSD checklist for DSM-5
PCR: Polymerase chain reaction
PHQ-15: Patient health questionnaire
PTSD: Post-traumatic stress disorder
RCI: Reliable change index
RCT: Randomised controlled trial
RT-PCR: Real time polymerase chain reaction
SAE: Serious adverse event
SARS: Severe acute respiratory syndrome
SES: Self-efficacy scale
SF-36: Short form health survey
SPIRIT: Standard protocol items: recommendations for interventional trials
SSL-D: Van Sonderen social support inventory subscale discrepancy
SSL-I: Van Sonderen Social Support Inventory subscale interactions
SD: Standard deviation
TMA: Transcription mediated amplification
WHO: World health organization
WSAS: Work and social adjustment scale
