# Supplementary material for: A randomised controlled trial testing the efficacy of Fit after COVID, a cognitive behavioural therapy targeting severe post-infectious fatigue following COVID-19 (ReCOVer): study protocol
Source: Trials. 2021 Dec 2;22:867. doi: 10.1186/s13063-021-05569-y (PMC8637041; doi:10.1186/s13063-021-05569-y)
Supplement: Supplementary file 1 — Additional file 1: Table S1. World Health Organization (WHO) Trial Registration Set. [file 13063_2021_5569_MOESM1_ESM.docx]

Online supplementary table 1 WHO Trial Registration Data Set

| Primary Registry and Trial Identifying Number | Dutch Trial Register (NTR)  NL8947 |
| --- | --- |
| Date of Registration in Primary Registry | 14 October 2020 |
| Secondary Identifying Numbers | NL74828.018.20 |
| Source(s) of Monetary or Material Support | ZonMW, grant nr.: 10430012010025 |
| Primary Sponsor | Prof. dr. J.A. Knoop,  Amsterdam University Medical Centers, Amsterdam Medical Center, Department of Medical Psychology  Amsterdam University Medical Centers, location Amsterdam Medical Center, Department of Medical Psychology  Location AMC  Department of Medical Psychology  Meibergdreef 9  1105 AZ Amsterdam  Email: [hans.knoop@amsterdamumc.nl](mailto:hans.knoop@amsterdamumc.nl)  Phone: +31 20 5667730 |
| Secondary Sponsor(s) | n.a. |
| Contact for Public Queries | T.A. Kuut  Department of Medical Psychology  Amsterdam University Medical Centers (VUmc)  PO BOX 7057  1007 MB Amsterdam  The Netherlands  e-mail: [t.kuut@amsterdamumc.nl](mailto:t.kuut@amsterdamumc.nl)  phone: +31 20 4443925 |
| Contact for Scientific Queries | Principal Investigator:  Prof. dr. J.A. Knoop,  Amsterdam University Medical Centers, location Amsterdam Medical Center, Department of Medical Psychology  Location AMC  Department of Medical Psychology  Meibergdreef 9  1105 AZ Amsterdam  Email: [hans.knoop@amsterdamumc.nl](mailto:hans.knoop@amsterdamumc.nl)  Phone: +31 20 5667730  T.A. Kuut  Department of Medical Psychology  Amsterdam University Medical Centers (VUmc)  PO BOX 7057  1007 MB Amsterdam  The Netherlands  e-mail: [t.kuut@amsterdamumc.nl](mailto:t.kuut@amsterdamumc.nl)  phone: +31 20 4443925 |
| Public Title | ReCOVer |
| Scientific Title | A Randomised Controlled Trial testing the efficacy of Fit after COVID, a Cognitive Behavioural Therapy targeting severe post-infectious fatigue following COVID-19 (ReCOVer) |
| Countries of Recruitment | The Netherlands |
| Health Condition(s) or Problem(s) Studied | Fatigue, COVID-19, Post-Acute Sequelae of SARS-CoV-2 Infection (PASC), post-COVID-19 syndrome, long COVID or Long-Haul COVID-19 Fatigue. |
| Intervention(s) | Interventions:  Fit after COVID: Cognitive behavioural therapy for severe fatigue following COVID-19.  Control arm: Care as usual. |
| Key Inclusion and Exclusion Criteria | **Inclusion criteria**   - Diagnosed with symptomatic COVID-19, confirmed by a positive PCR for SARS-CoV-2 *or* another positive NAAT test (RT-PCR, LAMP, TMA or mPOCT) *or* positive SARS-CoV-2 serology *or* a positive Antigen test *or* CORADS 4 or 5 on CT-scan. - 3 up to including 12 months after being diagnosed with COVID-19 or after hospital discharge in case the patient was admitted. - Severe fatigue, operationalised as a score ≥ 35 on the fatigue subscale of the Checklist Individual Strength (CIS). Fatigue started with or increased substantially directly after the onset of symptoms of COVID-19, as reported by the patient and confirmed by their GP or treating consultant. - Limitations in physical functioning operationalised as a score of ≤ 65 on the Short Form Health Survey (SF-36) or social disability operationalized as a score of ≥ 10 on the Work and Social Adjustment Scale (WSAS) - Age of 18 years or older. - Sufficient command of the Dutch language.   **Exclusion criteria**   - Known psychiatric or somatic condition that can explain the fatigue. Screening for somatic condition is done by the referring physician or the patient’s GP in case of self-referral. Participants are screened for the presence of Post-Traumatic Stress Disorder (PTSD) with the PTSD Checklist for DSM-5 (PCL-5) and for the presence of depressive disorder with the Beck Depression Inventory for Primary Care (BDI-PC). When the score on the BDI-PC ≥ 4 or score on the PCL-5 ≥ 33, the Mini-International Neuropsychiatric Interview (M.I.N.I.) is conducted to determine if patients meet the criteria of PTSD or a depressive disorder. - Current participation in a multi-disciplinary rehabilitation programme aimed to ameliorate the consequences of COVID-19. - Objective hypoxemia in rest for which oxygen therapy at home is indicated. |
| Study Type | A two-arm, multi-centre randomised controlled trial (RCT). |
| Date of First Enrolment | 12-11-2020 |
| Sample Size | Number of participants the trial plans to enrol: 114  Number of participants the trial has enrolled: 89 |
| Recruitment Status | Recruiting: participants are currently being recruited and enrolled. |
| Primary Outcome(s) | Fatigue severity, measured by the subscale Fatigue Severity of the Checklist Individual Strength (CIS-fatigue)  Time points:  T1 posttreatment or Care as usual  T2: follow-up, six months after T1 |
| Key Secondary Outcomes | The proportion of patients no longer being severely fatigued (operationalized in different ways)  Limitations in physical functioning, measured by the subscale Physical Functioning of the SF-36.  Social disability, measured by the Work and Social Adjustment Scale (WSAS) Somatic symptom severity, measured by the Somatic Symptoms scale of the Patient Health Questionnaire (PHQ).  Problems concentrating, measured by the concentration subscale of the CIS.  Time points:  T1 posttreatment or care as usual  T2: follow-up, six months after T1 |
| Ethics Review | Approved on 14-09-2020 by the Medical Ethical Committee of the Amsterdam University Medical Centers, Amsterdam University Medical Centers, location Amsterdam Medical Center (registration number 2020_182). |
| Expected completion date | Expected date of last inclusion: October 2021  Expected date of laste long term follow-up assessment: March 2023 |
| **IPD sharing statement** | Any request to share the data of this RCT will be considered by the trial steering committee and will need to be approved by the ethics committee of the Amsterdam University Medical Centers, location AMC. |
