# Supplementary material for: A randomised controlled trial testing the efficacy of Fit after COVID, a cognitive behavioural therapy targeting severe post-infectious fatigue following COVID-19 (ReCOVer): study protocol
Source: Trials. 2021 Dec 2;22:867. doi: 10.1186/s13063-021-05569-y (PMC8637041; doi:10.1186/s13063-021-05569-y)
Supplement: Supplementary file 2 — Additional file 2: Date and version identifier of the protocol. [file 13063_2021_5569_MOESM2_ESM.docx]

**Date and version identifier of the protocol**

Patient recruitment started in November 2020 with version 3 of the protocol. At the time of submission, 89 participants have been randomly assigned. The trial is being conducted in accordance with the protocol version 4, dated 21 April 2021.

Revision:

Version 1, date 27-07-2020

Version 2, date 01-09-2020: Questions of the medical ethics committee about the actigraph, AE’s and SAE’s, privacy and feasibility are answered. This information is added to the protocol.

Version 3, date 12-10-2020: the upper limit for including patients was extended from initially 6 months to 9 months after diagnosis or hospital discharge (inclusion criterion b).

Version 4, date 21-04-2021: the upper limit for including patients was extended from initially 6 months to 12 months after diagnosis or hospital discharge (inclusion criterion b); One of the original criteria to confirm COVID-19 (inclusion criterion a), i.e. typical symptoms and was part of a household in which another person was tested positive by PCR two weeks before or after the first day of illness, was removed because of the increased test capacity; new corona-virus tests are added to the inclusion criteria; CDC questionnaire is added to the T1 measurement.
