# Supplementary material for: A randomised controlled trial testing the efficacy of Fit after COVID, a cognitive behavioural therapy targeting severe post-infectious fatigue following COVID-19 (ReCOVer): study protocol
Source: Trials. 2021 Dec 2;22:867. doi: 10.1186/s13063-021-05569-y (PMC8637041; doi:10.1186/s13063-021-05569-y)
Supplement: Supplementary file 3 — Additional file 3: Informed consent form (in Dutch). [file 13063_2021_5569_MOESM3_ESM.pdf]

Proefpersoneninformatie voor deelname aan medisch-wetenschappelijk onderzoek

ReCOVer: Cognitieve gedragstherapie voor vermoeidheid na COVID-19

ReCOVer: A Randomised Controlled Trial testing the efficacy of Cognitive Behavioural Therapy for preventing chronic postinfectious fatigue among patients diagnosed with COVID-19

Inleiding

Geachte heer/mevrouw,

Wij vragen u om mee te doen aan een medisch-wetenschappelijk onderzoek. Meedoen is vrijwillig. Om mee te doen is wel uw schriftelijke toestemming nodig. U ontvangt deze brief omdat u eerder COVID-19 (een infectie met het coronavirus) heeft gehad.

Voordat u beslist of u wilt meedoen aan dit onderzoek, krijgt u uitleg over wat het onderzoek inhoudt. Lees deze informatie rustig door en vraag de onderzoeker uitleg als u vragen heeft. U kunt ook de onafhankelijk deskundige, die aan het eind van deze brief genoemd wordt, om aanvullende informatie vragen. U kunt er ook over praten met uw partner, vrienden of familie. Algemene informatie over meedoen aan zo'n onderzoek vindt u op de website van de Rijksoverheid: www.rijksoverheid.nl/mensenonderzoek.

1. Algemene informatie

Dit onderzoek is opgezet door Amsterdam UMC, locatie AMC, en wordt uitgevoerd door een team van onderzoekers, psychologen en artsen in verschillende ziekenhuizen en de GGD Amsterdam. Voor dit onderzoek zijn in totaal 114 proefpersonen uit Nederland nodig.
De medisch-ethische toetsingscommissie van Amsterdam UMC, locatie AMC heeft dit onderzoek goedgekeurd. Algemene informatie over de toetsing van onderzoek vindt u op de website van de Rijksoverheid: www.rijksoverheid.nl/mensenonderzoek.

2. Doel van het onderzoek

Het doel van dit onderzoek is uit te zoeken of cognitieve gedragstherapie ernstige vermoeidheid na het doormaken van COVID-19 vermindert. Beoogd wordt daarmee de ontwikkeling van chronische vermoeidheid te voorkomen.

3. Achtergrond van het onderzoek

Aanleiding van het onderzoek

Waarschijnlijk zal een aantal mensen die een infectie met het coronavirus (SARS CoV-2 ofwel COVID-19) heeft gehad, hiervan ook op lange termijn klachten ervaren. Naar verwachting hoort hier ook post-infectueuze chronische vermoeidheid bij; een klacht die vaak wordt gerapporteerd door mensen die infectieziekten hebben gehad. Mensen met post-infectueuze

chronische vermoeidheid zijn ernstig moe, langer dan 6 maanden, en zijn hierdoor beperkt in hun functioneren en deelname aan maatschappelijke activiteiten (bijvoorbeeld werk).

Omdat in de acute fase van COVID-19 vermoeidheid een veelvoorkomende klacht is, is de verwachting dat een deel van de mensen na COVID-19 te kampen krijgt met chronische post-COVID-19 vermoeidheid. Bij vermoeidheid na andere infectieziekten is gebleken dat cognitieve gedragstherapie de vermoeidheid effectief vermindert. Daarom willen we in deze studie uitzoeken of dit ook het geval is bij ernstige vermoeidheid na COVID-19. Door dit tijdig te doen willen we voorkomen dat de post-COVID-19 moeheid chronisch wordt.

Belang van het onderzoek

Het is belangrijk om te voorkomen dat mensen chronisch moe worden en beperkt blijven in hun functioneren. Wij verwachten dat een tijdige behandeling van de vermoeidheidsklachten na COVID-19 ertoe zal leiden dat mensen herstellen van de ernstige vermoeidheid, en minder mensen chronische klachten ontwikkelen. Dit is van belang voor individuele patiënten, en ook voor de maatschappij.

4. Wat meedoen inhoudt

Als u meedoet aan deze studie, duurt dat afhankelijk van welke groep u loot in totaal 10 of 16 maanden.

Screening

Wanneer u besluit om deel te nemen aan dit onderzoek, bepalen we eerst of u mee kunt doen. U krijgt een aantal korte vragenlijsten toegestuurd om te beoordelen of u aan de door ons gestelde criteria voldoet. Ook zullen we zo nodig met uw toestemming medische informatie bij uw specialist of huisarts opvragen uit de tijd dat bij u COVID-19 is vastgesteld. Het kan dus zijn dat op basis van de informatie uit de vragenlijsten u alsnog niet aan het onderzoek kunt deelnemen.

Behandeling

In dit onderzoek worden twee groepen met elkaar vergeleken. Door loting zal bepaald worden in welke groep u terechtkomt. De helft van de mensen wordt via loting toegewezen aan de interventiegroep, de andere helft aan de gebruikelijke zorg groep. In de interventiegroep wordt vermoeidheid behandeld met cognitieve gedragstherapie. In de gebruikelijke zorg groep krijgen mensen de gebruikelijke zorg, en mag 10 maanden na start van het onderzoek gestart worden met cognitieve gedragstherapie indien de klachten niet verminderd zijn en u behandeling wenst.

Cognitieve gedragstherapie is een behandeling waarbij verondersteld wordt dat u door uw denken en doen te veranderen, uw klachten kan verminderen. In wetenschappelijk onderzoek is aangetoond dat cognitieve gedragstherapie vermoeidheidsklachten effectief vermindert. In deze studie willen wij onderzoeken of ook vermoeidheid na COVID-19 effectief behandeld kan worden met cognitieve gedragstherapie, en of dit het ontwikkelen van chronische vermoeidheid (langer dan 6 maanden) kan voorkómen. De behandeling duurt gemiddeld 4 maanden.

Metingen en bezoeken

Metingen

Indien blijkt dat u kunt deelnemen aan het onderzoek, zullen er drie (gebruikelijke zorg groep) of vier (interventiegroep) metingen plaats vinden. Deze metingen zijn verspreid over 16 maanden:

- Bij start van het onderzoek (T0);
- 17 weken na de start van het onderzoek (T1);
- 10 maanden na de start van het onderzoek (T2);
- 16 maanden na de start van het onderzoek (T3), alleen als u de interventiegroep loot.

Deze metingen bestaan uit het invullen van een aantal vragenlijsten. Het invullen hiervan duurt de eerste keer (T0) ongeveer 45 minuten en daarop ongeveer 30 minuten per keer. Het invullen van de vragenlijsten kan vanuit huis gedaan worden (via het internet). Voor de mensen die gebruikelijke zorg volgen zal na de meting op T2 de behandeling starten indien zij dit wensen en zal er voor hen geen meting op T3 zijn.

Op T0 en T1 zullen wij u tevens vragen om gedurende 14 dagen een actigraph te dragen. De actigraph is een soort horloge die u om uw pols draagt en uw bewegingen meet. De actigraph draagt u dag en nacht gedurende de 14 dagen. De actigraph met bijbehorende instructie krijgt u thuisgestuurd via de post. Na afloop van de 14 dagen kunt u de actigraph terug sturen per post of meenemen naar de eerste afspraak met uw therapeut. De kosten die u maakt voor het terugsturen worden vergoed.

Daarnaast wordt u gevraagd om de periode dat u de actigraph draagt (14 dagen) tevens een slaapdagboek bij te houden. Het slaapdagboek is een papieren formulier waarop u dagelijks uw slaaptijden noteert. Het ingevulde slaapdagboek kunt u samen met de actigraph per post terugsturen.

Bezoeken

Door loting wordt bepaald in welke groep u terecht komt. Hieronder wordt per groep het aantal bezoeken in het ziekenhuis beschreven.

Gebruikelijke zorg groep:

Als u loot voor de gebruikelijke zorg groep, zult u gedurende maximaal 10 maanden de zorg krijgen zoals gebruikelijk is. U hoeft niet naar het ziekenhuis te komen. Bij de meting na 10 maanden, na het invullen van het derde vragenlijstpakket (T2), zullen uw vermoeidheidsklachten opnieuw geëvalueerd worden.

Indien u bij de meting na 10 maanden nog steeds klachten heeft en behoefte heeft aan zorg, zult u dan kunnen starten met cognitieve gedragstherapie. U heeft dan 1 gesprek met de therapeut op het ziekenhuis. De therapie wordt daarna via het internet gegeven en duurt 17 weken. Hierbij heeft u tussentijds contact met de therapeut via e-mail en/of videobellen.

Als u geen toegang heeft tot internet of geen internettherapie wilt dan kunt u voor de behandeling naar het ziekenhuis komen of kiezen voor videobellen. Deze behandeling bestaat dan uit maximaal 10 gesprekken met een therapeut. Een gesprek duurt ongeveer 45 minuten. De therapie wordt altijd gegeven door ervaren therapeuten verbonden aan het Amsterdam

UMC of een van de overige deelnemende ziekenhuizen. Het maakt daarbij niet uit of u de behandeling via het internet volgt, via videobellen of in het ziekenhuis.

Interventiegroep:
U vult de eerste vragenlijst thuis is. Als u loot voor de interventiegroep, zult u direct starten met cognitieve gedragstherapie. U komt minimaal één keer naar het ziekenhuis toe voor een kennismaking en intakegesprek met uw therapeut. De therapie wordt daarna via het internet gegeven en duurt 17 weken. Hierbij heeft u tussentijds contact met de therapeut via e-mail en/of videobellen.
Als u geen toegang heeft tot internet of geen internettherapie wilt dan kunt u voor de behandeling naar het ziekenhuis komen of kiezen voor videobellen. Deze behandeling bestaat dan uit maximaal 10 gesprekken met een therapeut. Een gesprek duurt ongeveer 45 minuten. De therapie wordt altijd gegeven door ervaren therapeuten verbonden aan het Amsterdam UMC of een van de overige deelnemende ziekenhuizen. Het maakt daarbij niet uit of u de behandeling via het internet volgt, via videobellen of in het ziekenhuis.

Anders dan bij gebruikelijke zorg
Wanneer u besluit om deel te nemen heeft dit geen invloed op eventuele medische zorg die u van uw behandelend specialist krijgt. De medische zorg blijft dus hetzelfde.

5. Wat wordt er van u verwacht

Om het onderzoek goed te laten verlopen, is het belangrijk dat u zich aan de volgende afspraken houdt.

De afspraken zijn dat u:

- op drie (of vier als u in de interventiegroep loot) momenten vragenlijsten invult;
- op twee momenten de actigraph draagt;
- op twee momenten een slaapdagboek bijhoudt;
- de cognitieve gedragstherapie volgt zoals deze wordt gegeven, en daarbij:
- afspraken voor bezoeken nakomt.

Het is belangrijk dat u contact opneemt met de onderzoeker:

- als u in een ziekenhuis wordt opgenomen;
- als u onder behandeling bent of gaat bij een psycholoog of psychiater;
- als u niet meer wilt meedoen aan het onderzoek;
- als uw contactgegevens wijzigen.

6. Mogelijke bijwerkingen en nadelige effecten

De te onderzoeken behandeling heeft zover wij weten geen bijwerkingen of andere nadelige effecten.

7. Mogelijke voor- en nadelen

Het is belangrijk dat u de mogelijke voor- en nadelen goed afweegt voordat u besluit mee te doen.

Voordelen van meedoen aan het onderzoek kunnen zijn:

- u krijgt behandeling voor vermoeidheid na COVID-19;
- aan de behandeling zijn geen kosten verbonden;
- u draagt bij aan het wetenschappelijk onderzoek naar effectieve behandeling van vermoeidheid na COVID-19.

Nadelen van meedoen aan het onderzoek kunnen zijn:

- u bent extra tijd kwijt aan het invullen van vragenlijsten;
- u draagt op twee momenten gedurende 14 dagen de actigraph;
- u houdt op twee momenten een slaapdagboek bij.

Al deze zaken zijn hiervoor onder punt 4, 5 en 6 beschreven.

8. Als u niet wilt meedoen of wilt stoppen met het onderzoek

U beslist zelf of u meedoet aan het onderzoek. Deelname is vrijwillig.

Als u niet wilt meedoen, wordt u op de gebruikelijke manier behandeld voor uw vermoeidheid na COVID-19. U vult dan waarschijnlijk minder vragenlijsten in. De onderzoeker kan u meer vertellen over de eventuele behandelmogelijkheden die er zijn en de voor- en nadelen daarvan.

Als u wel meedoet, kunt u zich altijd bedenken en toch stoppen, ook tijdens het onderzoek. U hoeft niet te zeggen waarom u stopt. Wel moet u dit direct melden aan de onderzoeker. De gegevens die tot dat moment zijn verzameld, worden gebruikt voor het onderzoek.

Als er nieuwe informatie over het onderzoek is die belangrijk voor u is, laat de onderzoeker dit aan u weten. U wordt dan gevraagd of u blijft meedoen.

9. Einde van het onderzoek

Uw deelname aan het onderzoek stopt als

- alle afspraken voor de behandeling voorbij zijn;
- u alle metingen heeft ingevuld;
- u zelf kiest om te stoppen;
- Amsterdam UMC, locatie AMC, de overheid of de beoordelende medisch-ethische toetsingscommissie, besluit om het onderzoek te stoppen.

Het hele onderzoek is afgelopen als alle deelnemers klaar zijn.

Na het verwerken van alle gegevens informeert de onderzoeker u over de belangrijkste uitkomsten van het onderzoek. Dit gebeurt ongeveer 12 maanden na uw deelname.

10. Gebruik en bewaren van uw gegevens

Voor dit onderzoek worden uw persoonsgegevens gebruikt en bewaard. Het gaat om gegevens zoals uw naam, adres, geboortedatum en om gegevens over uw gezondheid. Het verzamelen, gebruiken en bewaren van uw gegevens is nodig om contact met u op te nemen en om de vragen die in dit onderzoek worden gesteld te kunnen beantwoorden en de resultaten te kunnen publiceren.
Het is ook nodig om bepaalde persoonsgegevens, zoals uw naam en contactgegevens, in gebruikte applicaties in te voeren. Dit is nodig om vragenlijsten naar u toe te sturen en u een online behandeling aan te bieden. Uw persoonsgegevens hoeven niet in de actigraph worden ingevoerd. De actigraph slaat alleen gegevens over uw beweging op, welke gekoppeld zijn aan een code die niet direct te herleiden is tot uw persoonsgegevens.
Wij vragen voor het gebruik van uw gegevens uw toestemming.

Vertrouwelijkheid van uw gegevens

Om uw privacy te beschermen krijgen uw gegevens een code. Uw naam en andere gegevens die u direct kunnen identificeren worden daarbij weggelaten. Alleen met de sleutel van de code zijn gegevens tot u te herleiden. De sleutel van de code blijft veilig opgeborgen in de onderzoeksinstelling. Ook in rapporten en publicaties over het onderzoek zijn de gegevens niet tot u te herleiden.
Uw antwoorden op de vragenlijsten en in de online behandeling worden bewaard op een beveiligde server en zijn alleen toegankelijk voor geautoriseerd personeel (uw behandelaar en de onderzoekers van deze studie). Zoals hierboven genoemd, worden uw antwoorden onder een code bewaard.
Telefonische contacten vinden plaats via telefoons van het Amsterdam UMC. De telefonische contacten worden gevoerd in werkruimten van het Amsterdam UMC.

Toegang tot uw gegevens voor controle

Sommige personen kunnen op de onderzoekslocatie toegang krijgen tot al uw gegevens. Ook tot de gegevens zonder code. Dit is nodig om te kunnen controleren of het onderzoek goed en betrouwbaar is uitgevoerd. Personen die ter controle inzage krijgen in uw gegevens zijn: een controleur die voor Amsterdam UMC, locatie AMC werkt, en nationale toezichthoudende autoriteiten, bijvoorbeeld de Inspectie Gezondheidszorg en Jeugd. Zij houden uw gegevens geheim. Wij vragen u voor deze eventuele inzage toestemming te geven.

Bewaartermijn gegevens

Uw gegevens moeten 15 jaar worden bewaard bij de opdrachtgever (Amsterdam UMC, locatie AMC).

Bewaren en gebruik van gegevens voor ander onderzoek
Uw gegevens kunnen na afloop van dit onderzoek ook nog van belang zijn voor ander wetenschappelijk onderzoek op het gebied van de behandeling van post-infectueuze vermoeidheid of lange-termijn gevolgen van COVID-19. Daarvoor zullen uw gegevens 15 jaar worden bewaard. U kunt op het toestemmingsformulier aangeven of u hier wel of niet mee instemt. Indien u hier niet mee instemt, kunt u gewoon deelnemen aan het huidige onderzoek.

Intrekken toestemming
U kunt uw toestemming voor gebruik van uw persoonsgegevens altijd weer intrekken. Dit geldt voor dit onderzoek en ook voor het bewaren en het gebruik voor het toekomstige onderzoek. De onderzoeksgegevens die zijn verzameld tot het moment dat u uw toestemming intrekt worden nog wel gebruikt in het onderzoek.

Meer informatie over uw rechten bij verwerking van gegevens
Voor algemene informatie over uw rechten bij verwerking van uw persoonsgegevens kunt u de website van de Autoriteit Persoonsgegevens raadplegen.

Bij vragen over uw rechten kunt u contact opnemen met de verantwoordelijke voor de verwerking van uw persoonsgegevens. Voor dit onderzoek is dat:

Amsterdam UMC, locatie AMC. Zie bijlage A voor contactgegevens en website.

Bij vragen of klachten over de verwerking van uw persoonsgegevens raden we u aan eerst contact op te nemen met de onderzoekslocatie. U kunt ook contact opnemen met de Functionaris voor de Gegevensbescherming van de instelling (zie bijlage A voor de contactgegevens) of de Autoriteit Persoonsgegevens.

Registratie van het onderzoek
Informatie over dit onderzoek is ook opgenomen in een overzicht van medisch-wetenschappelijke onderzoeken, namelijk het Nederlands Trial Register. Daarin zijn geen gegevens opgenomen die naar u herleidbaar zijn. Na het onderzoek kan de website een samenvatting van de resultaten van dit onderzoek tonen. U vindt dit onderzoek in het Nederlands Trial Register onder de studienaam ‘ReCOVer’ nummer NL8947.

11. Verzekering voor proefpersonen

Als u deelneemt aan het onderzoek, loopt u geen extra risico’s. Amsterdam UMC, locatie AMC hoeft daarom van de medisch ethische toetsingscommissie (METc AMC) geen extra verzekering af te sluiten.

12. Informeren huisarts en/of behandelend specialist

Wij sturen uw huisarts en indien wenselijk uw behandelend specialist een brief om te laten weten dat u meedoet aan het onderzoek. Dit is voor de volledigheid van uw medisch dossier. Als u dit niet goed vindt, kunt u niet meedoen aan dit onderzoek.

13. Geen vergoeding voor meedoen

De behandeling voor het onderzoek kost u niets. U wordt niet betaald voor het meedoen aan dit onderzoek.

14. Heeft u vragen?

Bij vragen kunt u contact opnemen met het onderzoeksteam. Voor onafhankelijk advies over meedoen aan dit onderzoek kunt u terecht bij de onafhankelijke arts. Hij weet veel over het onderzoek, maar heeft niets te maken met dit onderzoek.
Indien u klachten heeft over het onderzoek, kunt u dit bespreken met de onderzoeker of uw behandelend arts. Wilt u dit liever niet, dan kunt u zich wenden tot de klachtenfunctionaris van het Amsterdam UMC, locatie AMC. Alle gegevens vindt u in **bijlage A**: Contactgegevens.

15. Ondertekening toestemmingsformulier

Wanneer u voldoende bedenktijd heeft gehad, wordt u gevraagd te beslissen over deelname aan dit onderzoek. Indien u toestemming geeft, zullen wij u vragen deze op de bijbehorende toestemmingsverklaring schriftelijk te bevestigen. Door uw schriftelijke toestemming geeft u aan dat u de informatie heeft begrepen en instemt met deelname aan het onderzoek.
Zowel uzelf als de onderzoeker ontvangen een getekende versie van deze toestemmingsverklaring.

Dank voor uw aandacht.

16. Bijlagen bij deze informatie

A. Contactgegevens

B. Toestemmingsformulier

C. Brochure 'Medisch Wetenschappelijk onderzoek. Algemene informatie voor de proefpersoon' (versie 01-03-2017)

Bijlage A: contactgegevens voor Amsterdam UMC, locatie AMC

Hoofdonderzoeker Amsterdam UMC, locatie AMC
Prof. dr. Hans Knoop
Amsterdam UMC, locatie AMC
Afdeling Medische Psychologie
Postbus 22660
1100 DD Amsterdam
Tel: 020-5667730
E-mail: hans.knoop@amsterdamumc.nl

Uitvoerend onderzoeker Amsterdam UMC, locatie AMC
Drs. Tanja Kuut
Amsterdam UMC, locatie VUmc
Afdeling Medische Psychologie
De Boelelaan 1118,
1081 HV Amsterdam
Tel: 020-4443925
E-mail: t.kuut@amsterdamumc.nl

Onafhankelijk arts:
Prof. dr. W. J. Wiersinga
Amsterdam UMC, location AMC
Inwendige geneeskunde, in het bijzonder translationele infectieziekten
Meibergdreef 9
1105 AZ Amsterdam
Tel: 020-561109
E-mail: w.j.wiersinga@amsterdamumc.nl

Klachten:
Afdeling Patiëntenvoorlichting en Klachtenopvang AMC
AMC Polikliniekgebouw, A0 (9.00-12.30 uur)
Postbus 22660
1100 DD Amsterdam
Tel: 020-5663355
E-mail: klachtenfunctionaris@amc.nl

Functionaris voor de Gegevensbescherming Amsterdam UMC, locatie AMC
Mr. Marleen Inge
fg@amc.nl

Voor meer informatie over uw rechten:
https://www.autoriteitpersoonsgegevens.nl

Bijlage B: toestemmingsformulier proefpersoon

ReCOVer: Cognitieve gedragstherapie voor vermoeidheid na COVID-19

- Ik heb de informatiebrief gelezen. Ook kon ik vragen stellen. Mijn vragen zijn voldoende beantwoord. Ik had genoeg tijd om te beslissen of ik meedoe.
- Ik weet dat meedoen vrijwillig is. Ook weet ik dat ik op ieder moment kan beslissen om toch niet mee te doen of te stoppen met het onderzoek. Daarvoor hoef ik geen reden te geven.
- Ik geef toestemming voor het informeren van mijn huisarts en indien wenselijk mijn behandelend specialist dat ik meedoe aan dit onderzoek.
- Ik geef toestemming voor het opvragen van informatie bij mijn huisarts/specialist(en) over mijn infectie met het coronavirus.
- Ik geef toestemming voor het verzamelen en gebruiken van mijn gegevens voor de beantwoording van de onderzoeksvraag in dit onderzoek.
- Ik weet dat voor de controle van het onderzoek sommige mensen toegang tot al mijn gegevens kunnen krijgen. Die mensen staan vermeld in deze informatiebrief. Ik geef toestemming voor die inzage door deze personen.
- Ik geef □ **wel**

  □ **geen**

  toestemming om mijn gegevens langer te bewaren en te gebruiken voor toekomstig onderzoek op het gebied van de behandeling van post-infectueuze vermoeidheid of lange-termijn gevolgen van COVID-19.
- Ik geef □ **wel**

  □ **geen**

  toestemming om mij na dit onderzoek opnieuw te benaderen voor een vervolgonderzoek.
- Ik wil □ **wel**

  □ **niet**

  na afloop van het onderzoek geïnformeerd worden over de resultaten van het onderzoek.
- Ik wil meedoen aan dit onderzoek.

Naam proefpersoon:

Handtekening: Datum : __ / __ / __

Alleen in te vullen door onderzoekers van de ReCOVer studie
Ik verklaar dat ik deze proefpersoon volledig heb geïnformeerd over het genoemde onderzoek.

Als er tijdens het onderzoek informatie bekend wordt die de toestemming van de proefpersoon zou kunnen beïnvloeden, dan breng ik hem/haar daarvan tijdig op de hoogte.

Naam onderzoeker (of diens vertegenwoordiger):
Handtekening: Datum: __ / __ / __

<indien van toepassing>
Aanvullende informatie is gegeven door:
Naam:
Functie:
Handtekening: Datum: __ / __ / __
----

* Doorhalen wat niet van toepassing is.

De proefpersoon krijgt een volledige informatiebrief mee, samen met een getekende versie van het toestemmingsformulier.
